# 1′,1′′-Dimethyl-4′-(naphthalen-1-yl)-1,2,3,4-tetra­hydro­naphthalene-2-spiro-3′-pyrrolidine-2′-spiro-3′′-indoline-1,2′′-dione

**DOI:** 10.1107/S1600536811007124

**Published:** 2011-03-02

**Authors:** S. Selvanayagam, K. Ravikumar, P. Saravanan, R. Raghunathan

**Affiliations:** a Department of Physics, Kalasalingam University, Krishnankoil 626 126, India; b Laboratory of X-ray Crystallography, Indian Institute of Chemical Technology, Hyderabad 500 007, India; c Department of Organic Chemistry, University of Madras, Guindy Campus, Chennai 600 025, India

## Abstract

In the title compound, C_32_H_28_N_2_O_2_, the pyrrolidine ring adopts an envelope conformation, whereas the cyclo­hexa­none ring in the tetra­hydro­naphthalene fused-ring system adopts a half-chair conformation. The oxindole ring system is oriented at an angle of 48.2 (1)° with respect to the naphthyl ring system. An intra­molecular C—H⋯O close contact is observed. In the crystal, mol­ecules associate *via* two C—H⋯O hydrogen bonds, forming *R*_2_^2^(14) and *R*_2_^2^(10) dimers.

## Related literature

For general background to pyrrolidine derivatives, see: Obniska *et al.* (2003 ▶); Peddi *et al.* (2004 ▶); Kaminski & Obniska (2008 ▶); Stylianakis *et al.* (2003 ▶). For related structures, see: Selvanayagam *et al.* (2011 ▶); Gans & Shalloway (2001 ▶). For ring-puckering parameters, see: Cremer & Pople (1975 ▶) and for asymmetry parameters, see: Nardelli (1983 ▶).
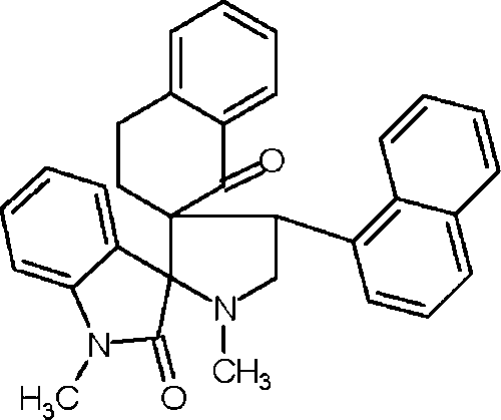


## Experimental

### 

#### Crystal data

C_32_H_28_N_2_O_2_
*M* *_r_* = 472.56
Monoclinic, 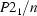

*a* = 8.7529 (8) Å
*b* = 18.0411 (16) Å
*c* = 15.4489 (13) Å
β = 98.181 (2)°
*V* = 2414.7 (4) Å^3^
*Z* = 4
Mo *K*α radiation
μ = 0.08 mm^−1^
*T* = 292 K
0.24 × 0.20 × 0.18 mm

#### Data collection

Bruker SMART APEX CCD area-detector diffractometer
27968 measured reflections
5745 independent reflections
4389 reflections with *I* > 2σ(*I*)
*R*_int_ = 0.030

#### Refinement

*R*[*F* ^2^ > 2σ(*F* ^2^)] = 0.055
*wR*(*F* ^2^) = 0.141
*S* = 1.04
5745 reflections
327 parameters
H-atom parameters constrained
Δρ_max_ = 0.27 e Å^−3^
Δρ_min_ = −0.18 e Å^−3^

### 

Data collection: *SMART* (Bruker, 2001 ▶); cell refinement: *SAINT* (Bruker, 2001 ▶); data reduction: *SAINT*; program(s) used to solve structure: *SHELXS97* (Sheldrick, 2008 ▶); program(s) used to refine structure: *SHELXL97* (Sheldrick, 2008 ▶); molecular graphics: *ORTEP-3* (Farrugia, 1997 ▶) and *PLATON* (Spek, 2009 ▶); software used to prepare material for publication: *SHELXL97* and *PLATON*.

## Supplementary Material

Crystal structure: contains datablocks I, global. DOI: 10.1107/S1600536811007124/ng5125sup1.cif

Structure factors: contains datablocks I. DOI: 10.1107/S1600536811007124/ng5125Isup2.hkl

Additional supplementary materials:  crystallographic information; 3D view; checkCIF report

## Figures and Tables

**Table 1:** Hydrogen-bond geometry (Å, °)

*D*—H⋯*A*	*D*—H	H⋯*A*	*D*⋯*A*	*D*—H⋯*A*
C12—H12*A*⋯O1	0.97	2.48	3.143 (2)	126
C13—H13*B*⋯O1^i^	0.97	2.58	3.482 (2)	156
C32—H32*A*⋯O1^ii^	0.96	2.59	3.364 (2)	138

Symmetry codes: (i) 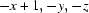; (ii) 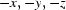.

**Fig. 1.**
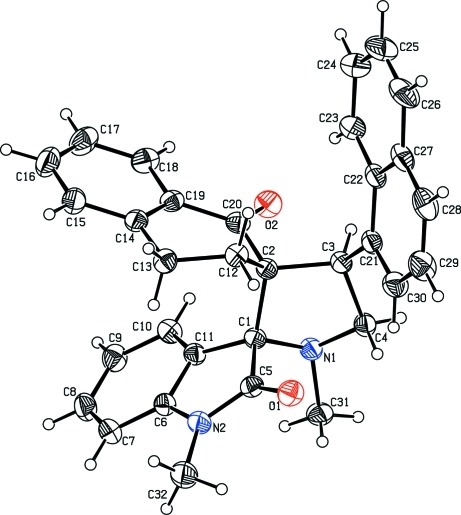
The molecular structure of the title compound, showing the atom-numbering scheme. Displacement ellipsoids are drawn at the 30% probability level

**Fig. 2.**
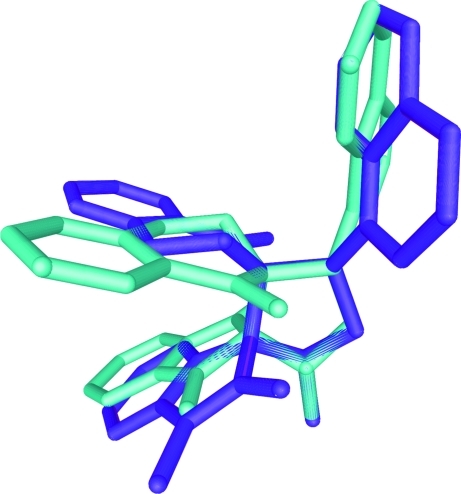
Superposition of (I) (violet) with the similar reported structure of Selvanayagam et al. (2011) (cyan).

**Fig. 3.**
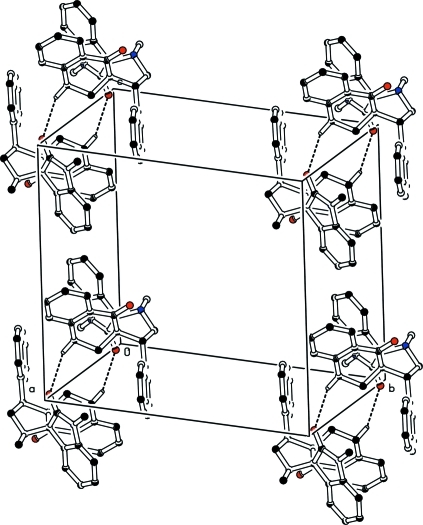
Molecular packing of the title compound, viewed along the a axis; H-bonds are shown as dashed lines forms a R22(14) dimers in unit cell. For the sake of clarity, H atoms, not involved in hydrogen bonds, have been omitted

**Fig. 4.**
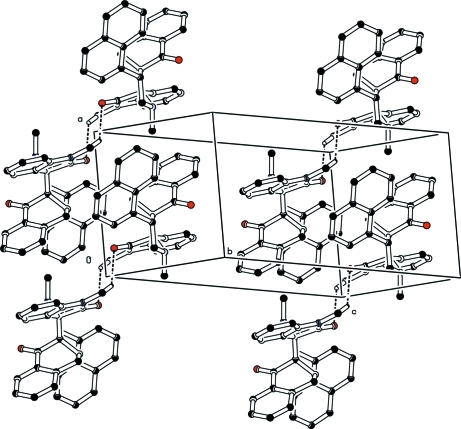
Molecular packing of the title compound, viewed down the b axis; H-bonds are shown as dashed lines forms a R22(10) dimers in unit cell. For the sake of clarity, H atoms, not involved in hydrogen bonds, have been omitted

## supplementary crystallographic information

### Comment

Spiro-pyrrolidine derivatives are unique tetracyclic 5-HT(2A) receptor antagonist (Obniska *et al.*, 2003; Peddi *et al.*, 2004). These derivatives possess anticonvulsant (Kaminski & Obniska, 2008) and anti-influenza virus (Stylianakis *et al.*, 2003) activities. In view of these importance and continuation of our work on the crystal structure analyis of spiro-pyrrolidine derivatives, we have undertaken the crystal structure determination of the title compound, and the results are presented here.

The X-ray study confirmed the molecular structure and atomic connectivity for (I), as illustrated in Fig. 1. The geometry of pyrrolidine, tetrahydro naphthalin and naphthyl group systems are comparable with the related reported structure (Selvanayagam *et al.*, 2011). Fig. 2 shows a superposition of the pyrrolidine ring of (I) with this related reported structure, using Qmol (Gans & Shalloway, 2001); the r.m.s. deviation is 0.363 Å.

The sum of the angles at N1 of the pyrrolidine ring [334.8°] and N2 of the oxindole ring [359.9°] are in accordance with sp^3^ and sp^2^ hybridizations. The short contacts H3···H23 (2.06 Å) and H4B···H30 (2 Å) result in substantial widening of the C21—C22—C23 and C21—C30—C29 bond angles [123.6 (2)° and 122.3 (2)°, respectively].

Pyrrolidine ring is in an envelope conformation, with puckering parameters q_2_ = 0.409 (1) Å and φ = -175.1 (2) °, and with atom N1 deviating -0.603 (2) Å from the least-squares plane passing through the remaining four atoms (C1-C4) of that ring (Cremer & Pople, 1975). The cyclohexanone ring in the tetrahydro naphthalin ring system has a half-chair conformation with the lowest asymmetry parameters of ΔC_2_(C2-C12) = 0.084 (1)° (Nardelli, 1983). The best plane of pyrrolidine ring system make a dihedral angles of 76.9 (1) and 68.9 (1)°, respectively with respect to the oxindole ring and naphtyl group systems.

The molecular structure is influenced by an intramolecular C—H···O close contacts. Atom O1 acts as a trifurcated acceptor for three intramolecular C—H···O contacts. In the molecular packing, C—H···O hydrogen bonds involving atoms C13 and O1 link inversion-related molecules to form R_2_^2^ (14) graph-set dimer (Fig. 3 and Table 1). In addition to this another graph-set dimer of R_2_^2^(10) forms in the unit cell involving C32 and O1 atoms via C-H···O hydrogen bonds (Fig. 4).

### Experimental

To a mixture of N-methyl isatin (1mmol), sarcosine (1mmol) and 2-napthalidene- 1,2,3,4-tetrahydronaphthalene-1-ones (1mmol) was added and heated under reflux in methanol (20ml) until the disappearance of the starting materials as evidenced by TLC. The solvent was removed under vacuo. The crude product was subjected to column chromatography using petroleum ether-ethyl acetate as eluent. Single crystals were grown by slow evaporation from methanol.

### Refinement

H atoms were placed in idealized positions and allowed to ride on their parent atoms, with C—H distances of 0.93-0.97 Å, and Uiso(H) = 1.5U_eq_(C) for methyl H and Uiso(H) = 1.2U_eq_(C) for all other H atoms.

### Crystal data

**Table:**

C_32_H_28_N_2_O_2_	*F*(000) = 1000
*M**_r_* = 472.56	*D*_x_ = 1.300 Mg m^−^^3^
Monoclinic, *P*2_1_/*n*	Mo *K*α radiation, λ = 0.71073 Å
Hall symbol: -P 2yn	Cell parameters from 17402 reflections
*a* = 8.7529 (8) Å	θ = 2.3–27.8°
*b* = 18.0411 (16) Å	µ = 0.08 mm^−^^1^
*c* = 15.4489 (13) Å	*T* = 292 K
β = 98.181 (2)°	Block, colourless
*V* = 2414.7 (4) Å^3^	0.24 × 0.20 × 0.18 mm
*Z* = 4	

### Data collection

**Table:**

Bruker SMART APEX CCD area-detector diffractometer	4389 reflections with *I* > 2σ(*I*)
Radiation source: fine-focus sealed tube	*R*_int_ = 0.030
graphite	θ_max_ = 28.0°, θ_min_ = 1.8°
ω scans	*h* = −11→11
27968 measured reflections	*k* = −23→23
5745 independent reflections	*l* = −20→19

### Refinement

**Table:**

Refinement on *F*^2^	Primary atom site location: structure-invariant direct methods
Least-squares matrix: full	Secondary atom site location: difference Fourier map
*R*[*F*^2^ > 2σ(*F*^2^)] = 0.055	Hydrogen site location: inferred from neighbouring sites
*wR*(*F*^2^) = 0.141	H-atom parameters constrained
*S* = 1.04	*w* = 1/[σ^2^(*F*_o_^2^) + (0.0661*P*)^2^ + 0.5875*P*] where *P* = (*F*_o_^2^ + 2*F*_c_^2^)/3
5745 reflections	(Δ/σ)_max_ = 0.001
327 parameters	Δρ_max_ = 0.27 e Å^−^^3^
0 restraints	Δρ_min_ = −0.18 e Å^−^^3^

### Special details

**Table:**

Geometry. All esds (except the esd in the dihedral angle between two l.s. planes) are estimated using the full covariance matrix. The cell esds are taken into account individually in the estimation of esds in distances, angles and torsion angles; correlations between esds in cell parameters are only used when they are defined by crystal symmetry. An approximate (isotropic) treatment of cell esds is used for estimating esds involving l.s. planes.
Refinement. Refinement of F^2^ against ALL reflections. The weighted R-factor wR and goodness of fit S are based on F^2^, conventional R-factors R are based on F, with F set to zero for negative F^2^. The threshold expression of F^2^ > 2sigma(F^2^) is used only for calculating R-factors(gt) etc. and is not relevant to the choice of reflections for refinement. R-factors based on F^2^ are statistically about twice as large as those based on F, and R- factors based on ALL data will be even larger.

### Fractional atomic coordinates and isotropic or equivalent isotropic displacement parameters (Å^2^)

**Table:**

	*x*	*y*	*z*	*U*_iso_*/*U*_eq_	
O1	0.19542 (14)	0.03811 (7)	0.02474 (7)	0.0503 (3)	
O2	0.51135 (15)	0.18921 (7)	0.29060 (8)	0.0526 (3)	
N1	0.17919 (14)	0.13236 (7)	0.18988 (8)	0.0394 (3)	
N2	0.20315 (16)	−0.04854 (7)	0.13326 (9)	0.0447 (3)	
C1	0.28805 (16)	0.07193 (8)	0.17953 (9)	0.0352 (3)	
C2	0.43644 (16)	0.11610 (8)	0.16072 (9)	0.0341 (3)	
C3	0.36697 (17)	0.19082 (8)	0.11804 (10)	0.0375 (3)	
H3	0.4055	0.2308	0.1583	0.045*	
C4	0.19270 (19)	0.18552 (9)	0.12043 (11)	0.0440 (4)	
H4A	0.1507	0.2333	0.1336	0.053*	
H4B	0.1390	0.1680	0.0649	0.053*	
C5	0.22581 (17)	0.02033 (9)	0.10128 (10)	0.0393 (3)	
C6	0.24558 (18)	−0.05153 (9)	0.22415 (11)	0.0410 (4)	
C7	0.2350 (2)	−0.11139 (10)	0.27874 (13)	0.0528 (4)	
H7	0.2003	−0.1573	0.2567	0.063*	
C8	0.2781 (2)	−0.10037 (11)	0.36793 (13)	0.0558 (5)	
H8	0.2723	−0.1397	0.4063	0.067*	
C9	0.3292 (2)	−0.03231 (10)	0.40053 (12)	0.0517 (4)	
H9	0.3571	−0.0261	0.4605	0.062*	
C10	0.33951 (19)	0.02735 (9)	0.34430 (10)	0.0436 (4)	
H10	0.3735	0.0733	0.3665	0.052*	
C11	0.29901 (17)	0.01766 (8)	0.25571 (10)	0.0377 (3)	
C12	0.53834 (18)	0.07288 (9)	0.10482 (10)	0.0386 (3)	
H12A	0.4776	0.0604	0.0492	0.046*	
H12B	0.6227	0.1044	0.0930	0.046*	
C13	0.60428 (18)	0.00205 (9)	0.14878 (10)	0.0431 (4)	
H13A	0.5209	−0.0326	0.1530	0.052*	
H13B	0.6743	−0.0206	0.1132	0.052*	
C14	0.68899 (17)	0.01682 (9)	0.23853 (10)	0.0417 (4)	
C15	0.8018 (2)	−0.03200 (11)	0.27723 (13)	0.0542 (4)	
H15	0.8222	−0.0751	0.2480	0.065*	
C16	0.8835 (2)	−0.01708 (13)	0.35840 (14)	0.0660 (6)	
H16	0.9577	−0.0505	0.3836	0.079*	
C17	0.8563 (2)	0.04692 (13)	0.40262 (12)	0.0637 (5)	
H17	0.9136	0.0571	0.4567	0.076*	
C18	0.7440 (2)	0.09559 (11)	0.36647 (11)	0.0524 (4)	
H18	0.7249	0.1385	0.3965	0.063*	
C19	0.65839 (17)	0.08082 (9)	0.28461 (10)	0.0401 (4)	
C20	0.53440 (17)	0.13366 (9)	0.24958 (10)	0.0382 (3)	
C21	0.41452 (18)	0.21033 (9)	0.03005 (10)	0.0406 (4)	
C22	0.55816 (19)	0.24838 (8)	0.02709 (11)	0.0427 (4)	
C23	0.6600 (2)	0.26977 (10)	0.10265 (12)	0.0518 (4)	
H23	0.6329	0.2604	0.1577	0.062*	
C24	0.7977 (2)	0.30391 (12)	0.09610 (16)	0.0671 (6)	
H24	0.8623	0.3179	0.1465	0.080*	
C25	0.8418 (3)	0.31796 (13)	0.01394 (18)	0.0758 (7)	
H25	0.9361	0.3405	0.0100	0.091*	
C26	0.7475 (3)	0.29878 (12)	−0.05961 (16)	0.0697 (6)	
H26	0.7779	0.3087	−0.1137	0.084*	
C27	0.6039 (2)	0.26398 (9)	−0.05623 (12)	0.0514 (4)	
C28	0.5078 (3)	0.24300 (11)	−0.13333 (12)	0.0621 (5)	
H28	0.5378	0.2531	−0.1875	0.075*	
C29	0.3724 (3)	0.20835 (11)	−0.12918 (12)	0.0635 (5)	
H29	0.3092	0.1952	−0.1804	0.076*	
C30	0.3263 (2)	0.19202 (10)	−0.04740 (12)	0.0531 (4)	
H30	0.2327	0.1680	−0.0461	0.064*	
C31	0.02001 (18)	0.10912 (10)	0.19308 (12)	0.0496 (4)	
H31A	−0.0432	0.1520	0.1976	0.074*	
H31B	0.0168	0.0777	0.2430	0.074*	
H31C	−0.0180	0.0823	0.1407	0.074*	
C32	0.1366 (3)	−0.10906 (10)	0.07871 (14)	0.0646 (6)	
H32A	0.0785	−0.0895	0.0263	0.097*	
H32B	0.0696	−0.1375	0.1100	0.097*	
H32C	0.2176	−0.1403	0.0637	0.097*	

### Atomic displacement parameters (Å^2^)

**Table:**

	*U*^11^	*U*^22^	*U*^33^	*U*^12^	*U*^13^	*U*^23^
O1	0.0545 (7)	0.0563 (7)	0.0389 (6)	−0.0025 (6)	0.0017 (5)	−0.0018 (5)
O2	0.0589 (8)	0.0525 (7)	0.0462 (7)	0.0012 (6)	0.0059 (6)	−0.0133 (5)
N1	0.0365 (7)	0.0373 (7)	0.0465 (7)	0.0007 (5)	0.0127 (6)	0.0009 (6)
N2	0.0472 (8)	0.0365 (7)	0.0483 (8)	−0.0041 (6)	−0.0007 (6)	−0.0035 (6)
C1	0.0357 (7)	0.0356 (8)	0.0349 (7)	−0.0018 (6)	0.0076 (6)	−0.0006 (6)
C2	0.0347 (7)	0.0349 (7)	0.0336 (7)	−0.0014 (6)	0.0078 (6)	0.0007 (6)
C3	0.0393 (8)	0.0339 (7)	0.0398 (8)	−0.0011 (6)	0.0074 (6)	0.0011 (6)
C4	0.0404 (8)	0.0389 (8)	0.0540 (10)	0.0030 (7)	0.0109 (7)	0.0065 (7)
C5	0.0349 (7)	0.0417 (8)	0.0414 (8)	0.0003 (6)	0.0055 (6)	−0.0030 (6)
C6	0.0383 (8)	0.0369 (8)	0.0477 (9)	0.0009 (6)	0.0060 (7)	0.0013 (7)
C7	0.0543 (10)	0.0360 (9)	0.0670 (12)	−0.0017 (7)	0.0053 (9)	0.0053 (8)
C8	0.0585 (11)	0.0490 (10)	0.0606 (11)	0.0035 (8)	0.0110 (9)	0.0193 (9)
C9	0.0549 (10)	0.0589 (11)	0.0424 (9)	0.0032 (8)	0.0101 (8)	0.0100 (8)
C10	0.0478 (9)	0.0441 (9)	0.0407 (8)	−0.0018 (7)	0.0127 (7)	−0.0006 (7)
C11	0.0358 (7)	0.0373 (8)	0.0412 (8)	−0.0002 (6)	0.0098 (6)	0.0032 (6)
C12	0.0403 (8)	0.0439 (8)	0.0330 (7)	0.0014 (7)	0.0096 (6)	−0.0012 (6)
C13	0.0420 (8)	0.0433 (9)	0.0455 (9)	0.0052 (7)	0.0112 (7)	−0.0032 (7)
C14	0.0347 (7)	0.0479 (9)	0.0440 (8)	−0.0020 (7)	0.0103 (6)	0.0086 (7)
C15	0.0466 (9)	0.0540 (11)	0.0623 (11)	0.0049 (8)	0.0088 (8)	0.0130 (9)
C16	0.0513 (11)	0.0806 (15)	0.0640 (13)	0.0110 (10)	0.0009 (9)	0.0251 (11)
C17	0.0533 (11)	0.0942 (16)	0.0406 (9)	−0.0003 (10)	−0.0035 (8)	0.0144 (10)
C18	0.0491 (10)	0.0690 (12)	0.0391 (9)	−0.0066 (9)	0.0060 (7)	0.0030 (8)
C19	0.0352 (8)	0.0506 (9)	0.0352 (8)	−0.0050 (7)	0.0076 (6)	0.0051 (7)
C20	0.0379 (8)	0.0431 (8)	0.0351 (8)	−0.0056 (6)	0.0105 (6)	−0.0010 (6)
C21	0.0448 (9)	0.0344 (8)	0.0432 (8)	0.0011 (6)	0.0084 (7)	0.0069 (6)
C22	0.0469 (9)	0.0325 (8)	0.0506 (9)	0.0018 (7)	0.0134 (7)	0.0079 (7)
C23	0.0502 (10)	0.0502 (10)	0.0562 (10)	−0.0067 (8)	0.0115 (8)	0.0060 (8)
C24	0.0557 (12)	0.0652 (13)	0.0798 (14)	−0.0152 (10)	0.0076 (10)	0.0036 (11)
C25	0.0618 (13)	0.0721 (14)	0.0978 (18)	−0.0219 (11)	0.0264 (13)	0.0141 (13)
C26	0.0814 (15)	0.0554 (12)	0.0805 (15)	−0.0124 (11)	0.0400 (13)	0.0132 (11)
C27	0.0641 (11)	0.0369 (9)	0.0573 (11)	0.0010 (8)	0.0227 (9)	0.0107 (7)
C28	0.0898 (15)	0.0540 (11)	0.0460 (10)	0.0008 (10)	0.0212 (10)	0.0144 (8)
C29	0.0851 (15)	0.0614 (12)	0.0419 (10)	−0.0082 (11)	0.0014 (9)	0.0077 (9)
C30	0.0572 (11)	0.0540 (10)	0.0474 (10)	−0.0074 (8)	0.0049 (8)	0.0083 (8)
C31	0.0378 (8)	0.0542 (10)	0.0597 (11)	−0.0030 (7)	0.0165 (8)	0.0017 (8)
C32	0.0726 (13)	0.0447 (10)	0.0697 (13)	−0.0067 (9)	−0.0137 (10)	−0.0104 (9)

### Geometric parameters (Å, °)

**Table:**

O1—C5	1.2175 (19)	C14—C15	1.393 (2)
O2—C20	1.2179 (19)	C14—C19	1.402 (2)
N1—C4	1.456 (2)	C15—C16	1.379 (3)
N1—C31	1.4626 (19)	C15—H15	0.9300
N1—C1	1.4715 (19)	C16—C17	1.379 (3)
N2—C5	1.362 (2)	C16—H16	0.9300
N2—C6	1.401 (2)	C17—C18	1.376 (3)
N2—C32	1.450 (2)	C17—H17	0.9300
C1—C11	1.523 (2)	C18—C19	1.401 (2)
C1—C5	1.561 (2)	C18—H18	0.9300
C1—C2	1.585 (2)	C19—C20	1.487 (2)
C2—C12	1.539 (2)	C21—C30	1.369 (2)
C2—C20	1.544 (2)	C21—C22	1.439 (2)
C2—C3	1.584 (2)	C22—C23	1.418 (2)
C3—C21	1.519 (2)	C22—C27	1.429 (2)
C3—C4	1.534 (2)	C23—C24	1.370 (3)
C3—H3	0.9800	C23—H23	0.9300
C4—H4A	0.9700	C24—C25	1.401 (3)
C4—H4B	0.9700	C24—H24	0.9300
C6—C7	1.381 (2)	C25—C26	1.351 (3)
C6—C11	1.396 (2)	C25—H25	0.9300
C7—C8	1.390 (3)	C26—C27	1.413 (3)
C7—H7	0.9300	C26—H26	0.9300
C8—C9	1.378 (3)	C27—C28	1.409 (3)
C8—H8	0.9300	C28—C29	1.349 (3)
C9—C10	1.394 (2)	C28—H28	0.9300
C9—H9	0.9300	C29—C30	1.411 (3)
C10—C11	1.375 (2)	C29—H29	0.9300
C10—H10	0.9300	C30—H30	0.9300
C12—C13	1.522 (2)	C31—H31A	0.9600
C12—H12A	0.9700	C31—H31B	0.9600
C12—H12B	0.9700	C31—H31C	0.9600
C13—C14	1.500 (2)	C32—H32A	0.9600
C13—H13A	0.9700	C32—H32B	0.9600
C13—H13B	0.9700	C32—H32C	0.9600
			
C4—N1—C31	113.04 (13)	C15—C14—C19	118.54 (16)
C4—N1—C1	106.63 (11)	C15—C14—C13	120.75 (16)
C31—N1—C1	115.21 (13)	C19—C14—C13	120.70 (14)
C5—N2—C6	111.54 (13)	C16—C15—C14	120.73 (19)
C5—N2—C32	122.92 (15)	C16—C15—H15	119.6
C6—N2—C32	125.49 (15)	C14—C15—H15	119.6
N1—C1—C11	111.27 (12)	C15—C16—C17	120.67 (18)
N1—C1—C5	111.46 (12)	C15—C16—H16	119.7
C11—C1—C5	101.04 (12)	C17—C16—H16	119.7
N1—C1—C2	101.98 (11)	C18—C17—C16	119.80 (18)
C11—C1—C2	120.09 (12)	C18—C17—H17	120.1
C5—C1—C2	111.26 (11)	C16—C17—H17	120.1
C12—C2—C20	108.06 (12)	C17—C18—C19	120.30 (19)
C12—C2—C3	114.61 (12)	C17—C18—H18	119.8
C20—C2—C3	109.09 (12)	C19—C18—H18	119.8
C12—C2—C1	113.80 (12)	C18—C19—C14	119.92 (16)
C20—C2—C1	107.81 (11)	C18—C19—C20	118.39 (15)
C3—C2—C1	103.18 (11)	C14—C19—C20	121.69 (14)
C21—C3—C4	115.86 (13)	O2—C20—C19	120.29 (14)
C21—C3—C2	115.60 (12)	O2—C20—C2	121.28 (14)
C4—C3—C2	105.14 (12)	C19—C20—C2	118.42 (13)
C21—C3—H3	106.5	C30—C21—C22	118.29 (15)
C4—C3—H3	106.5	C30—C21—C3	122.29 (15)
C2—C3—H3	106.5	C22—C21—C3	119.42 (14)
N1—C4—C3	104.12 (12)	C23—C22—C27	117.62 (16)
N1—C4—H4A	110.9	C23—C22—C21	123.62 (15)
C3—C4—H4A	110.9	C27—C22—C21	118.73 (16)
N1—C4—H4B	110.9	C24—C23—C22	121.23 (18)
C3—C4—H4B	110.9	C24—C23—H23	119.4
H4A—C4—H4B	109.0	C22—C23—H23	119.4
O1—C5—N2	124.73 (15)	C23—C24—C25	120.4 (2)
O1—C5—C1	126.79 (14)	C23—C24—H24	119.8
N2—C5—C1	108.42 (13)	C25—C24—H24	119.8
C7—C6—C11	122.27 (16)	C26—C25—C24	120.1 (2)
C7—C6—N2	127.64 (15)	C26—C25—H25	119.9
C11—C6—N2	110.07 (13)	C24—C25—H25	119.9
C6—C7—C8	117.45 (16)	C25—C26—C27	121.5 (2)
C6—C7—H7	121.3	C25—C26—H26	119.2
C8—C7—H7	121.3	C27—C26—H26	119.2
C9—C8—C7	121.21 (16)	C28—C27—C26	121.06 (18)
C9—C8—H8	119.4	C28—C27—C22	119.87 (17)
C7—C8—H8	119.4	C26—C27—C22	119.06 (19)
C8—C9—C10	120.45 (17)	C29—C28—C27	120.49 (17)
C8—C9—H9	119.8	C29—C28—H28	119.8
C10—C9—H9	119.8	C27—C28—H28	119.8
C11—C10—C9	119.44 (16)	C28—C29—C30	120.26 (19)
C11—C10—H10	120.3	C28—C29—H29	119.9
C9—C10—H10	120.3	C30—C29—H29	119.9
C10—C11—C6	119.16 (15)	C21—C30—C29	122.34 (18)
C10—C11—C1	131.76 (14)	C21—C30—H30	118.8
C6—C11—C1	108.89 (13)	C29—C30—H30	118.8
C13—C12—C2	112.84 (12)	N1—C31—H31A	109.5
C13—C12—H12A	109.0	N1—C31—H31B	109.5
C2—C12—H12A	109.0	H31A—C31—H31B	109.5
C13—C12—H12B	109.0	N1—C31—H31C	109.5
C2—C12—H12B	109.0	H31A—C31—H31C	109.5
H12A—C12—H12B	107.8	H31B—C31—H31C	109.5
C14—C13—C12	111.68 (13)	N2—C32—H32A	109.5
C14—C13—H13A	109.3	N2—C32—H32B	109.5
C12—C13—H13A	109.3	H32A—C32—H32B	109.5
C14—C13—H13B	109.3	N2—C32—H32C	109.5
C12—C13—H13B	109.3	H32A—C32—H32C	109.5
H13A—C13—H13B	107.9	H32B—C32—H32C	109.5
			
C4—N1—C1—C11	172.75 (12)	C5—C1—C11—C6	−1.96 (15)
C31—N1—C1—C11	−60.95 (17)	C2—C1—C11—C6	−124.64 (14)
C4—N1—C1—C5	−75.29 (15)	C20—C2—C12—C13	57.40 (16)
C31—N1—C1—C5	51.01 (17)	C3—C2—C12—C13	179.26 (12)
C4—N1—C1—C2	43.50 (14)	C1—C2—C12—C13	−62.30 (17)
C31—N1—C1—C2	169.80 (12)	C2—C12—C13—C14	−54.08 (17)
N1—C1—C2—C12	−151.75 (12)	C12—C13—C14—C15	−156.85 (15)
C11—C1—C2—C12	84.77 (16)	C12—C13—C14—C19	21.9 (2)
C5—C1—C2—C12	−32.81 (17)	C19—C14—C15—C16	−1.1 (3)
N1—C1—C2—C20	88.40 (13)	C13—C14—C15—C16	177.67 (17)
C11—C1—C2—C20	−35.07 (17)	C14—C15—C16—C17	−0.7 (3)
C5—C1—C2—C20	−152.65 (12)	C15—C16—C17—C18	1.6 (3)
N1—C1—C2—C3	−26.95 (13)	C16—C17—C18—C19	−0.7 (3)
C11—C1—C2—C3	−150.43 (13)	C17—C18—C19—C14	−1.1 (2)
C5—C1—C2—C3	91.99 (13)	C17—C18—C19—C20	177.82 (16)
C12—C2—C3—C21	−1.88 (18)	C15—C14—C19—C18	2.0 (2)
C20—C2—C3—C21	119.41 (14)	C13—C14—C19—C18	−176.80 (14)
C1—C2—C3—C21	−126.16 (13)	C15—C14—C19—C20	−176.92 (14)
C12—C2—C3—C4	127.22 (14)	C13—C14—C19—C20	4.3 (2)
C20—C2—C3—C4	−111.49 (13)	C18—C19—C20—O2	1.8 (2)
C1—C2—C3—C4	2.94 (15)	C14—C19—C20—O2	−179.27 (14)
C31—N1—C4—C3	−169.85 (13)	C18—C19—C20—C2	−178.04 (13)
C1—N1—C4—C3	−42.26 (16)	C14—C19—C20—C2	0.9 (2)
C21—C3—C4—N1	151.51 (13)	C12—C2—C20—O2	149.50 (14)
C2—C3—C4—N1	22.57 (16)	C3—C2—C20—O2	24.30 (19)
C6—N2—C5—O1	−178.51 (15)	C1—C2—C20—O2	−87.09 (17)
C32—N2—C5—O1	−1.1 (3)	C12—C2—C20—C19	−30.67 (17)
C6—N2—C5—C1	−1.40 (17)	C3—C2—C20—C19	−155.87 (13)
C32—N2—C5—C1	176.01 (15)	C1—C2—C20—C19	92.74 (15)
N1—C1—C5—O1	60.76 (19)	C4—C3—C21—C30	−28.9 (2)
C11—C1—C5—O1	179.05 (15)	C2—C3—C21—C30	94.74 (19)
C2—C1—C5—O1	−52.3 (2)	C4—C3—C21—C22	152.08 (14)
N1—C1—C5—N2	−116.28 (14)	C2—C3—C21—C22	−84.27 (18)
C11—C1—C5—N2	2.01 (15)	C30—C21—C22—C23	−179.50 (16)
C2—C1—C5—N2	130.63 (13)	C3—C21—C22—C23	−0.4 (2)
C5—N2—C6—C7	178.27 (17)	C30—C21—C22—C27	−1.3 (2)
C32—N2—C6—C7	1.0 (3)	C3—C21—C22—C27	177.77 (14)
C5—N2—C6—C11	0.08 (18)	C27—C22—C23—C24	−0.2 (3)
C32—N2—C6—C11	−177.24 (16)	C21—C22—C23—C24	178.00 (18)
C11—C6—C7—C8	0.8 (3)	C22—C23—C24—C25	−0.6 (3)
N2—C6—C7—C8	−177.22 (16)	C23—C24—C25—C26	0.9 (4)
C6—C7—C8—C9	0.1 (3)	C24—C25—C26—C27	−0.4 (4)
C7—C8—C9—C10	−0.3 (3)	C25—C26—C27—C28	−178.8 (2)
C8—C9—C10—C11	−0.4 (3)	C25—C26—C27—C22	−0.4 (3)
C9—C10—C11—C6	1.2 (2)	C23—C22—C27—C28	179.18 (16)
C9—C10—C11—C1	175.58 (15)	C21—C22—C27—C28	0.9 (2)
C7—C6—C11—C10	−1.4 (2)	C23—C22—C27—C26	0.7 (3)
N2—C6—C11—C10	176.88 (14)	C21—C22—C27—C26	−177.61 (16)
C7—C6—C11—C1	−177.00 (15)	C26—C27—C28—C29	178.6 (2)
N2—C6—C11—C1	1.31 (17)	C22—C27—C28—C29	0.1 (3)
N1—C1—C11—C10	−58.3 (2)	C27—C28—C29—C30	−0.7 (3)
C5—C1—C11—C10	−176.78 (16)	C22—C21—C30—C29	0.8 (3)
C2—C1—C11—C10	60.5 (2)	C3—C21—C30—C29	−178.26 (17)
N1—C1—C11—C6	116.47 (14)	C28—C29—C30—C21	0.2 (3)

### Hydrogen-bond geometry (Å, °)

**Table:**

*D*—H···*A*	*D*—H	H···*A*	*D*···*A*	*D*—H···*A*
C3—H3···O2	0.98	2.25	2.783 (2)	113
C4—H4B···O1	0.97	2.49	3.045 (2)	116
C12—H12A···O1	0.97	2.48	3.143 (2)	126
C32—H32A···O1	0.96	2.52	2.852 (2)	100
C13—H13B···O1^i^	0.97	2.58	3.482 (2)	156
C32—H32A···O1^ii^	0.96	2.59	3.364 (2)	138

Symmetry codes: (i) −*x*+1, −*y*, −*z*; (ii) −*x*, −*y*, −*z*.
